# Does Timing of Completion Radical Cholecystectomy Determine the Survival Outcome in Incidental Carcinoma Gallbladder: A Single-Center Retrospective Analysis

**DOI:** 10.7759/cureus.26653

**Published:** 2022-07-08

**Authors:** Rahul Rahul, Kulbhushan Haldenia, Ashish Singh, Vishwas Kapoor, Rajneesh K Singh, Rajan Saxena

**Affiliations:** 1 Department of Surgical Gastroenterology, Sanjay Gandhi Postgraduate Institute of Medical Sciences, Lucknow, IND; 2 Department of Biostatistics & Health Informatics, Sanjay Gandhi Postgraduate Institute of Medical Sciences, Lucknow, IND

**Keywords:** timing, survival, re-resection, gallbladder cancer, incidental

## Abstract

Introduction

Incidental discovery of gallbladder cancer (GBC) on postoperative histopathology or intra-operative suspicion is becoming increasingly frequent since laparoscopic cholecystectomy became the standard of care for gallstone disease. Incidental GBC (IGBC) portends a better survival than primarily detected GBC. Various factors affect the outcome of re-resection with the timing of re-intervention an important determinant of survival.

Methods

All patients of IGBC who underwent curative resection from January 2009 to December 2018 were considered for analysis. Details of demographic profile, index surgery, and operative findings on re-resection, histopathology and follow-up were retrieved from the prospectively maintained database. Patients were evaluated in three groups based on the interval between index cholecystectomy and re-resection: Early (<4 weeks), Intermediate (4-12 weeks) and Late (>12 weeks), using appropriate statistical tests.

Results

Ninety-one patients were admitted with IGBC during the study period of which 48 underwent re-resection with curative intent. The median age of presentation was 55 years (31-77 years). The median duration of follow-up was 40.6 months (Range: 1.2-130.6 months). Overall and disease-free survival among the above-mentioned three groups was the best in the early group (104 and 102 months) as compared to the intermediate (84 and 83 months) and late groups (75 and 73 months), though the difference failed to achieve statistical significance (p=0.588 and 0.581). On univariate analysis, factors associated with poor outcome were node metastasis, need for common bile duct (CBD) excision and high-grade tumor. However, on multivariate analysis, poor differentiation was the only independent factor affecting survival.

Conclusion

Early surgery, preferably within four weeks, possibly entails better survival in incidentally detected GBC. The grade of a tumor, however, is the most important determinant of survival in IGBC.

## Introduction

Gall bladder carcinoma (GBC) accounts for 85-90% of all biliary tract cancers and displays a marked ethnic, geographic and gender variation in terms of prevalence. It is more common (2-5 times) in females across all age groups, regions and ethnicity. The incidence rate is high among Asians (India, Japan and China), South Americans (Chile, Peru and Ecuador) and residents of few European countries (Spain, Germany and Slovakia). The geographical and gender variation coincides with the variation in the incidence of gall bladder (GB) stones. A selective predilection for GBC in a certain population indicates the possibility of genetic predisposition [1,2]. Other risk factors associated with the development of GBC include increasing age, cigarette smoking, anomalous pancreaticobiliary junction, choledochal cysts, chronic Salmonella infection, solitary large GB polyp and porcelain GB [2].

Traditionally, GBC has been associated with a dismal prognosis with a five-year overall survival rate less than 10%. Patients usually present in an advanced stage due to a lack of pathognomonic symptoms in the initial phase of the disease [3,4]. The majority (50-80%) of patients with a primary diagnosis of GBC are not considered surgical candidates due to local or distant advanced disease [5,6]. In the recent past, with the increasing popularity of laparoscopic cholecystectomy, incidental discovery of GBC on postoperative histopathology or intra-operative suspicion is becoming increasingly frequent. A true incidental GBC (IGBC) is defined as the detection of malignancy on histopathology with no suspicion in the preoperative or intraoperative period. This carries a good prognosis as the disease is usually in its early stage. Unfortunately, only a small subset (<1%) of patients falls in this category. In the majority of cases, the disease is discovered or suspected during intra-abdominal exploration based on operative findings and/or on gross examination of the specimen. Another subset of patients (either histopathology not done or histopathology inconclusive) presents with multiple metastasis few months following index cholecystectomy. Inadequate preoperative workup or inexperience on the part of treating surgeon is a possible cause. Authors have variably described such an encounter as unexpected or missed GBC [7,8].

Most of the patients with true IGBC merit an evaluation for re-resection. Completion radical surgery in a select group can achieve remarkable results in terms of survival outcome. One of the important factors affecting survival is the timing of re-intervention. While an early surgery is advocated to decrease the chances of dissemination, an intentional delay of three months has been suggested by few for biological staging [9,10]. The present study was conducted to evaluate the factors influencing survival in incidental GBC including the timing of re-intervention. This article was previously presented as Free Oral paper at the 2021 Liver Week in Korea on May 13th, 2021.

## Materials and methods

Patient and methods

In 10 years (January 2009 to December 2018), 91 patients with IGBC were admitted in the surgical gastroenterology ward, of which 48 could undergo curative resection. Patients with Stage 1 disease on index cholecystectomy (T1a) or metastatic disease on imaging, laparoscopy, or exploration and those who did not consent for surgery were not considered for curative resection. Progress reports, demographic and operative details were accessed through a prospectively maintained hospital database. Follow-up of data (follow-up cards, telephonic or through messages) was maintained till December 2019 which included subsequent histopathology report, adjuvant therapy and overall/disease-free survival. The work has been reported in line with the STROCSS (Strengthening the reporting of cohort studies in surgery) criteria [11]. This retrospective study is registered on ‘Clinicaltrials.gov - Protocol Registration and Results System’ (Registration number - NCT05114369).

Tumors were staged as per the eighth edition of AJCC-TNM staging. All the patients were evaluated by contrast-enhanced axial imaging (CECT - contrast-enhanced computed tomography) to stage the disease. Positron emission tomography (PET) scan was added to rule out distant disease in patients who presented beyond 12 weeks or in presence of high-risk features on CECT (residual disease or lymph node). Clinically fit patients with no evidence of distant metastasis on imaging were considered for staging laparoscopy (to rule out ascites, peritoneal or omental nodules and serosal deposits). It was preferably done in patients presenting in intermediate or late period and those with evidence of residual tumor in the gallbladder bed. Inter-aortocaval (IAC) tissue was then sent for frozen section analysis. Once IAC node was reported free of tumor, radical re-resection was attempted which included wedge resection of the liver or segment 4b/5 excision with regional lymphadenectomy. Extrahepatic bile duct excision was added only if the cystic duct margin was found positive for malignancy or in presence of direct invasion into the duct. Port site excision is not a part of our institutional protocol.

All available histopathology blocks and slides following the index cholecystectomy were re-reviewed by the pathologists at our institute. Tumor characteristics and primary staging were documented. Interval between index cholecystectomy and date of re-operation was calculated for all the patients and divided into three groups: Early (E) (<4 weeks), Intermediate (I) (4-12 weeks) and Late (L) (>12 weeks). In the present study, we intend to assess the effect of tumor stage, tumor characteristics and time gap (between index surgery and re-resection) on overall survival following curative (R0/R1) resection.

Statistical analysis

Median (range) represented continuous variables, while frequency (%) was used to express categorical data. A variable was considered to be under Gaussian distribution when the Z-score was within ±3.29 (n≥50). Independent sample t-test/Mann-Whitney U test was used to compare the mean/median among groups, and Chi-square/Fisher test was used for categorical data. Kaplan-Meier survival plots represented the survival data. We conducted a Cox regression analysis to identify the predictors of survival. Variables found significant in univariate analysis were included for multivariate analysis as well. All analyses were conducted considering a two-tailed p-value of <0.05 as statistically significant. Statistical Package for the Social Sciences version-23 (SPSS-23, IBM Corp., Armonk, NY, USA) and MedCalc software were used for data analysis.

## Results

During the study period, 48 patients [11 (22.9%) patients in Group ‘E’, 31 (64.5%) in Group ‘I’ and six (12.5%) patients in Group ‘L’] of IGBC (all referred from outside) underwent curative re-resection. The demography (age and gender), co-morbid status (diabetes, hypertension, coronary artery disease, asthma, etc.), tumor stage and characteristics, presence or absence of residual disease, extent of resection, resection margin, node retrieval, final stage of malignancy and adjuvant therapy were similar in the three groups (each p>0.05). The median age was 55 years with the disease being 2.7 times more common among females (Tables 1, 2).

**Table 1:** Distribution of clinical characteristics of the patients as per their timing of treatment of curative re-resection (N=48) Data presented in Frequency (%), compared by Fisher exact test. p<0.05 is significant. *Data not available for 12 patients. CTRT: Chemoradiotherapy; SSI: Surgical Site Infection; pT: pathological tumor stage

Variables	Early (E) N=11 (%)	Intermediate (I) N=31 (%)	Late (L) N=6 (%)	Total N=48 (%)	P-value
Residual Disease after Surgery	5 (45.45)	12 (38.71)	3 (50)	20 (41.67)	0.347
Differentiation of Tumor*					
Grade 1	3 (27.27)	7 (22.58)	2 (33.33)	12(25.0)	0.788
Grade 2	4 (36.36)	20 (64.51)	3 (50.0)	27 (56.25)	
Grade 3	0 (0)	2 (6.45)	0 (0)	2 (4.17)	
Primary stage of the tumor (following index cholecystectomy)					
pT1b	1 (9.09)	3 (9.67)	2 (33.33)	6 (12.50)	0.555
pT2	8 (72.72)	20 (64.51)	3 (50.0)	31 (64.58)	
pT3	2 (18.18)	8 (25.80)	1 (16.67)	11 (22.92)	
Final stage after curative resection					
Stage 1	1 (9.09)	1 (3.22)	2 (33.33)	4 (8.32)	0.225
Stage 2	6 (54.54)	19 (61.29)	2 (33.33)	27 (56.25)	
Stage 3	4 (36.36)	8 (25.80)	2 (33.33)	14 (29.16)	
Stage 4	0 (0)	3 (9.67)	0 (0)	3 (6.25)	
Adjuvant Therapy					
At least one cycle	5 (45.45)	21 (67.74)	3 (50.0)	29 (60.41)	0.380
≥2 cycles	5 (45.45)	21 (67.74)	2 (33.33)	28 (58.33)	0.209
CTRT	3 (27.27)	15 (48.38)	2 (33.33)	20 (41.67)	0.519
Morbidity					
≥ ClaveinDindo Grade 3	1 (9.09)	2 (6.45)	0 (0)	3 (6.25)	0.418
SSI	1 (9.09)	5 (16.13)	2 (33.33)	8 (16.67)	0.456
Bile Leak	1 (9.09)	1 (3.22)	0 (0)	2 (4.16)	0.587
Chyle Leak	0 (0)	0 (0)	1 (16.67)	1 (2.08)	0.125
Intra-abdominal collection	0 (0)	3 (9.67)	0 (0)	3 (6.25)	0.704

**Table 2:** Distribution of demographic and clinical characteristics of the patients in three groups (N=48) Data presented in Median (Range), compared by Kruskal Wallis H test. Frequency (%), compared by Fisher exact test. Mean±SD, compared by One Way ANOVA test. p<0.05 significant CCX: Cholecystectomy; CBD: Common bile duct

Variables	Early (E) N=11 (%)	Intermediate (I) N=31 (%)	Late (L) N=6 (%)	Total N=48 (%)	p-value
Age (Median, Range)	55 (32-74)	55 (32-77)	48 (31-57)	55 (31-77)	0.269
Sex (Male)	4 (36.36)	7 (22.58)	2 (33.33)	13 (27.1)	0.552
Comorbidities					
0	5 (45.45)	18 (58.06)	5 (83.33)	28 (58.33)	0.154
1	6 (54.54)	9 (29.03)	0 (0)	15 (31.25)	
2	0 (0)	4 (12.90)	1 (16.67)	5 (10.41)	
Resection Margin					
R0	11 (100)	29 (93.55)	5 (83.33)	45 (93.75)	0.998
R1	0 (0)	2 (6.45)	1 (16.67)	3 (12.5)	
Surgery – Type of Radical CCX					
Wedge Resection	10 (90.91)	25 (80.64)	4 (66.66)	39 (81.25)	0.447
Seg 4b,5	1 (9.09)	1 (3.23)	1 (16.67)	3 (6.25)	
Wedge resection with CBD excision	0 (0)	5 (16.13)	1 (16.67)	6 (12.5)	
Average number of nodes	8.7±3.7	10±4.4	8.7±6	9.5±4.4	0.626
Node positivity (No of patients with positive LN)	2 (18.18)	6 (19.35)	1 (16.67)	9 (18.75)	0.166

No postoperative mortality was recorded. The most common complication in the recovery period was surgical site infection (SSI). Three in the 'I' group required percutaneous drainage for intra-abdominal collection. No patient during the study period required re-exploration (Table 2).

Overall survival (OS) and disease-free survival (DFS)

Mean and median follow-up was 51.6 and 40.6 months, respectively (range: 1.2-130.6). A total of nine (18.7%) patients were reported lost to follow up during the study period. Mean OS and DFS in the study cohort were 91.75 (95% CI: 8.32-75.44) and 85.63 (95% CI: 69.48-101.79) months respectively (As the patient OS and DFS percentage did not reach <50%, the resultant median survival could not be computed).

In our study, 29 (60.4%) of those undergoing definitive surgery received adjuvant chemotherapy. Around 58% received >1 cycle and 44% could complete their therapy.

**Figure 1:**
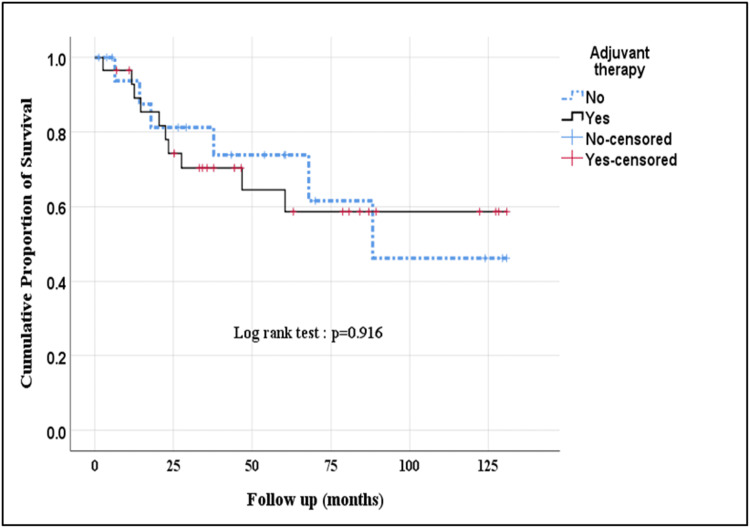
Overall impact of adjuvant therapy on event-free survival

Radiotherapy was given in 42% of patients. The most important indications for adjuvant therapy included good performance status with advanced stage and margin positive and/or node-positive disease. Difference in OS [88.10 (95% CI: 67.18-109.03) Vs 87.56 (95% CI: 61.66-113.45) months (p=0.916) and DFS [86.18 (95% CI: 65.73-106.64) Vs 85.37 (95% CI: 59.82-110.91) months (p=0.915) among patients who received adjuvant therapy was not statistically significant as compared to those who were not offered chemotherapy (Figure 1).

**Figure 2:**
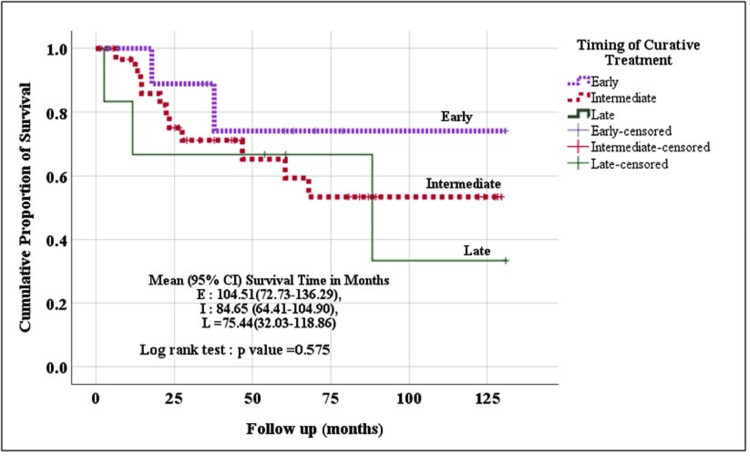
Overall event-free survival in study patients in the three groups

**Figure 3:**
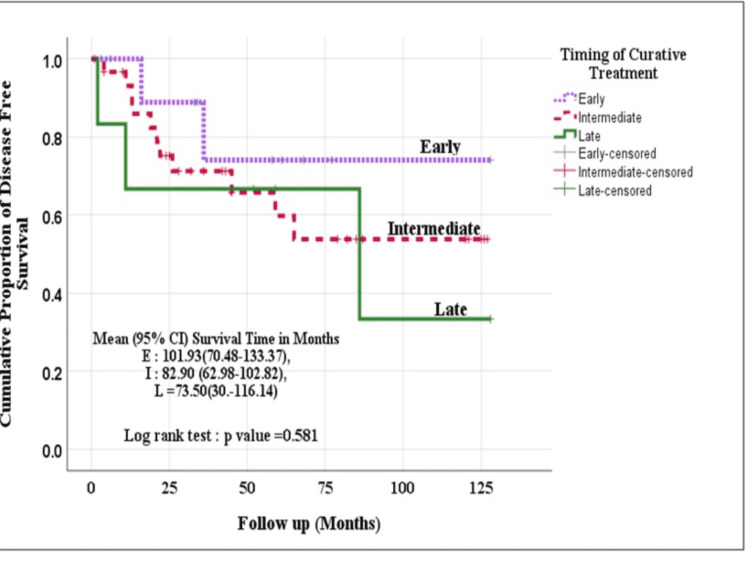
Disease-free survival in study patients as per the timing of treatment

Kaplan Meier method (log-rank test) was used to evaluate the survival (OS and DFS) among the three groups. Though the patients operated in the early (within four weeks) and intermediate period (4-12 weeks) fared better than those operated in the late period (beyond 12 weeks), the difference was not statistically significant [Event-free survival time (p=0.575) and disease-free survival time (p=0.581)] (Figures 2, 3).

Survival analysis

On univariate analysis of the factors affecting the outcome of curative surgery, nodal metastasis, requirement of common bile duct (CBD) excision and higher grade of tumor were associated with significantly poor survival. In addition, the presence of residual disease in the gallbladder bed, lymphovascular invasion and surgery beyond 12 weeks of index cholecystectomy were harbingers of poor survival outcome, although the difference was statistically insignificant. Residual tumor was present in 20 out of 48 (41.6%) patients. The probability of finding residual disease activity in re-resected specimen increased with higher T-stage on initial biopsy (16% in T1, 29% in T2 and 72% in T3 tumors). Primary tumor stage, resection margin and use of adjuvant therapy had little or no effect on survival outcome (each p>0.05) (Table 3).

**Table 3:** Univariate analysis of predictors of overall survival following curative resection * Mean survival as more than 50% were alive at last follow-up. CBD: Common bile duct; R: Resection status; T: Tumor stage

Variables	Hazard ratio (95% CI)	P-Value	Mean*OS (months)
Primary Stage		0.937	
T1	Reference		89.50
T2	1.32 (0.29-6.08)	0.719	82.54
T3	1.25 (0.23-6.83)	0.799	84.97
Node status			
Node +	Reference	0.016	46.44
Node -	0.27 (0.09-0.78)		92.82
R status			
R0	0.90 (0.12-6.85)	0.918	87.50
R1	Reference		63.58
Time of re-resection		0.588	
Early	0.39 (0.07-2.37)	0.308	104.51
Intermediate	0.75 (0.21-2.70)	0.658	84.65
Late	Reference		75.44
PNI/LVI status			
Positive	Reference	0.366	64.25
Negative	0.56 (0.16-1.97)		90.77
Grade of Tumor		0.021	
Well diff	0.06 (0.008-0.477)	0.008	104.83
Moderately diff	0.14 (0.027-0.708)	0.018	82.03
Poorly diff	Reference		14.88
Adjuvant Therapy			
Yes	0.947 (0.34-2.61)	0.916	88.11
No	Reference		87.56
Extent of resection		0.038	
Wedge resection	0.26 (0.08-0.83)	0.023	98.51
Seg 4b/5 resection	0.92 (0.17-5.10)	0.927	53.05
Extended Cholecystectomy + CBD excision	Reference		19.13
Final stage of disease			
Stage 1 and 2	0.83 (0.30-2.28)	0.713	88.01
Stage 3 and 4	Reference		83.24
Residual disease			
Yes	Reference		71.27
No	0.41 (0.15-1.10)	0.078	98.84

To further assess the predictors of survival among the participants, multivariate Cox regression analysis was used. Three variables (Disease spread to nodes, Tumor grades and Extent of resection) found significant on Univariate analysis were subjected to multivariate analysis. Higher tumor grade was the only significant independent predictor of survival. The presence of positive nodes (p=0.052) and the need for CBD excision (0.133) were associated with the increased relative risk of disease-related mortality, although the difference was not statistically significant (Table 4).

**Table 4:** Multivariate analysis of predictors of the survival of the patients following Curative resection (N=48) Multivariate Cox regression analysis used. P<0.05 significant CBD: Common bile duct

Variables	Hazard Ratio			P-value
	Value	95% Confidence Interval		
		Lower	Upper	
Node status				
Node +	Reference			0.053
Node -	0.29	0.08	1.02	
Grade of Tumor				0.012
Well diff	0.07	0.009	0.587	0.014
Moderately diff	0.07	0.011	0.440	0.005
Poorly diff	Reference			
Extent of resection				0.133
Wedge resection	0.26	0.07	1.01	0.052
Segment 4b/5 resection	0.80	0.08	8.10	0.850
Extended Cholecystectomy + CBD excision	Reference			

## Discussion

Cholecystectomy is one of the commonest procedures in general surgery. The wide use of minimal invasive techniques has made the procedure extremely popular. Nearly 0.3-3% of the specimens present with a histological surprise (presence of malignancy) [12-13]. Almost all surgeons performing cholecystectomy encounter IGBC at least once in their professional carrier. The incidence of IGBC is continuously rising. At present 40-60% of all GBC is incidentally detected [14,15]. The majority of them merit evaluation for re-resection. Current recommendations for radical surgery are largely based on the T-stage (T1b, T2 and T3) following index cholecystectomy. The presence of a positive cystic lymph node also sets up an indication for re-surgery, although not a reliable indicator as skip metastasis to regional lymph nodes without the involvement of the cystic node is common in GBC. Moreover, dissection in the Calot’s triangle during cholecystectomy starts lateral to the cystic node and is often not a part of the specimen. The probability of (locoregional) residual disease increases with the T-stage. The reported incidence is 12-50% with T1 tumors, 31-66% with T2 tumors and 46-80% with T3 tumors. The presence of residual disease and higher T-stage are associated with poor survival [16-20]. In the present study, the residual disease was found in 16%, 29% and 72% of T1b, T2 and T3 disease respectively on re-resection. The presence of residual tumor was associated with poor survival (71 Vs 98 months), though the difference could not achieve statistical significance.

Apart from primary T-stage, factors affecting OS following re-resection include lymph node status, resection margin, lymphovascular invasion, grade of tumor and timing of intervention. Pawlik et al. reported a five-year survival rate of 73% in the absence of nodal metastasis against 27% in the presence of nodal disease following completion radical cholecystectomy [16]. Ouchi et al. documented poor survival in the presence of high-grade tumors with lymphovascular invasion in GBC [21]. In a report by Butte et al., the histologic grade of tumors was the strongest determinant of survival following re-resection [20]. A higher grade has been associated with the presence of metastasis and early recurrence [20,22]. In the current series, survival in node-positive disease and poorly differentiated tumors following re-resection was significantly less. The presence of lymphovascular invasion was also associated with worse survival (64 Vs 90 months), though the difference could not achieve statistical significance.

Resection margin status (R0/R1) did not affect the survival, possibly due to the addition of chemoradiotherapy in all margin positive resections. Moreover, the number of R1 resections was low (3/48). Ethun et al., in their study, demonstrated poor survival associated with advanced T-stage and R2 resection. No difference was documented between R0/R1 resection [10].

In our study, wedge resection alone was performed in the majority (81%) of patients. Nearly 6% underwent segment 4b/5 resection and CBD excision was done in 13%. None of the patients underwent major hepatic resection. The extent of resection was guided by the tumor infiltration or cystic margin status on the frozen section, with the aim of achieving R0 resection. On Univariate analysis, patients requiring CBD excision demonstrated a poor survival as compared to patients undergoing liver resection alone (19 months for CBD excision Vs 53 and 98 months for Seg 4b and 5 resection and wedge resection respectively). However, on multivariate analysis, the difference could not achieve statistical significance, although the requirement of extended surgeries portended poor survival. Similar findings were documented by Fuks et al., who emphasized on the importance of complete resection, rather than the extent of surgery (segmental resection versus non-anatomical wedge resection). Bile duct resection in both T2 and T3 disease in the series was associated with poor survival [17]. This can be explained by the fact that the involvement of CBD in itself is an indication of an advanced tumor and is more likely to disseminate to distant nodes.

The debate on the timing of reoperation revolves around the quest between technical ease of dissection (adhesions and inflammation in the early postoperative period) and biological selection (aggressive tumors tend to present with obvious metastasis with time). The question yet to be answered is the optimum time when an aggressive tumor should be operated on. The usual practice is to intervene as soon as possible. Many retrospective studies have compared the effect of time interval on the final outcome. Ethun et al. divided the patients who were re-operated at 10 high volume centers into three groups: <4 weeks, 4-8 weeks and beyond eight weeks. The majority of the patients were operated between 4-8 weeks. There was no difference in T-stage, grade of tumor, resection rate and residual disease in the three groups, yet the highest survival was documented in the intermediate group (40 months). The author concluded that subclinical advanced disease may take more than eight weeks to manifest themselves [10]. Another group from Britain deliberately delayed the evaluation for re-resection by 12 weeks from the date of index surgery and the radical surgery was performed in 49% of the cohort. Overall median survival was 20.7 months (54.8 months in those who could undergo re-resection). This study lacked a control group. Though the authors could avoid unnecessary laparotomy but for one (2%), the median survival of 20.7 months for the entire cohort was much below the existing international standards [9]. A contrasting report from China stressed on intervention within two weeks. The authors divided the patients into three groups: <2 weeks, 2-4 weeks and >4 weeks. The survival in the early group was significantly more than in the other two groups (86 months Vs 27 months). No technical difficulty in early surgery was reported [23]. Concurring with the Chinese study, the OS in the present series was better in the early (104 months) and intermediate groups (84 months) as compared to the late group (75 months) (p=0.588). Similarly, the DFS following curative resection fared better in the early (102 months) and intermediate groups (83 months) than the late group (73 months) though the difference was not statistically significant (p=0.581). A larger cohort or a longer follow-up could have clearly established the statistical significance. The average duration between index cholecystectomy and successful re-operation is usually ~7 weeks across various reports [16,22]. The authors do not recommend a delay in evaluation and intervention. More than half of the patients in our study were operated on within 4-12 weeks. This is the usual time of presentation as the majority of cholecystectomies are performed in the periphery. Suspecting a benign disease to start with, the pace of reporting the histopathology is usually slow. Further arrangement for logistics and transport in a developing country like India is often difficult. These factors along with the stress of heavy patient load on public sector hospitals often result in an undue delay in the final treatment. We suggest evaluation for treatment as early as possible for IGBC as it confers increased OS.

The addition of adjuvant therapy was not associated with improved survival (88 Vs 87 months) on Univariate analysis. Nearly 60% of the patients with good performance status and high-risk factors (high-grade tumor, advanced disease, node metastasis, residual disease and margin positive resection) received adjuvant therapy in the present series. The addition of adjuvant treatment apparently mitigates the effects of these adverse/high-risk attributes. Indications of postoperative chemoradiotherapy in IGBC have been extrapolated from that of primary GBC. The SEER (Surveillance, Epidemiology, and End Results) database documented the benefits of chemoradiation over chemotherapy alone in the selected group of GBC patients [24]. The chance of tumor breach/bile spill during the initial surgery is an additional factor that justifies the use of adjuvant treatment in IGBC to prevent early recurrence [25]. As the patients of IGBC often undergo an index cholecystectomy at a remote center, the sequence of intra-operative events remains elusive. In such situations, it is prudent to keep the threshold of additional therapy low.

Being a retrospective analysis, detailed information regarding index procedure (bile spill, use of bag for extraction of the specimen, breach of tumor margin) was missing or incomplete. This was a single-center study which has its own advantages and disadvantages. While the variation in protocol was minimal and follow-up was robust, we had to compromise on the number of recruits even when the study period was 10 years long.

## Conclusions

Survival in IGBC can be improved by early intervention (preferably within four weeks), although a significant difference could not be reached due to the small sample size. The most important tumor factor affecting survival is the tumor grade (high grade associated with poor survival, 14 months Vs >82 months). The presence of nodal metastasis, higher primary T-stage, presence of residual disease in the gallbladder bed and requirement of extensive resection (CBD excision) have a negative impact on overall survival. The final stage of the disease or use of adjuvant therapy has little bearing on the survival outcome. Validation of the facts entails a controlled study on a larger cohort.
